# Network analysis of Tourette syndrome and attention-deficit/hyperactivity disorder symptoms in children and adolescents

**DOI:** 10.1186/s13034-024-00810-3

**Published:** 2024-09-16

**Authors:** Wenyan Zhang, Zhongliang Jiang, Anyi Zhang, Liping Yu, Xianbin Wang, Xu Hong, Yonghua Cui, Tianyuan Lei

**Affiliations:** 1 Department of Psychiatry, Beijing Children’s Hospital, Capital Medical University, National Center for Children’s Health, Beijing, 100045 China; 2 Laboratory for Clinical Medicine, Capital Medical University, Beijing, 100045 China; 3 Cloud Services Innovation Laboratory, Institute of Intelligent Science and Technology, Electronics Technology Group Corporation, 100041 Beijing, China

**Keywords:** Tourette syndrome, Attention-deficit/hyperactivity disorder, Network analysis, Children and adolescents

## Abstract

**Background:**

While Tourette syndrome (TS) and attention-deficit/hyperactivity disorder (ADHD) often co-occur, the nature of the relationship between their symptoms is not well understood. Network analysis of psychopathology allow for detailed examinations of symptom interactions, providing an effective approach to explore the patterns of comorbidity between TS and ADHD symptoms.

**Methods:**

This study included 3,958 participants (male/female = 3,004/954, age mean ± SD = 8.60 ± 2.25 years). We collected data on TS symptoms using the Motor Tic, Obsessions and Compulsions, Vocal Tic Evaluation Survey (MOVES), and ADHD symptoms using the Swanson, Nolan, and Pelham Rating Scale-IV (SNAP-IV). Network analysis was employed to construct a combined network of TS and ADHD symptoms at the symptom level. We utilized the expected influence (EI) and bridge EI metrics to explore the core and bridge symptoms within the network.

**Results:**

The network structure demonstrated a moderate number of non-zero connections between TS and ADHD symptoms, constituting 23.06% of all potential connections. Core symptoms in the comorbidity network included “Often has difficulty sustaining attention in tasks or play activities,” “Certain bad words or thoughts keep going through my mind,” and “Words come out that I can’t stop or control.” Bridging symptoms identified were “Words come out that I can’t stop or control,” “I do certain things like jumping or clapping over and over,” “I can’t control all my movements,” and “Often talks excessively.”

**Conclusion:**

The core and bridging symptoms identified in this study serve as potential therapeutic targets for the treatment of TS and ADHD comorbidity in clinical children and adolescents.

**Supplementary Information:**

The online version contains supplementary material available at 10.1186/s13034-024-00810-3.

## Introduction

Tourette syndrome (TS) and attention-deficit/hyperactivity disorder (ADHD) are prevalent persistent neurodevelopmental disorders in children. TS is characterized by frequent motor and vocal tics persisting for over a year [1], whereas ADHD is primarily marked by symptoms of inattention, impulsivity, and hyperactivity [2]. In community samples of children and adolescents, the prevalence of TS is about 1% [3], while ADHD is more common, with rates ranging from 2 to 7%, averaging around 5% [4]. These disorders often co-occur, with ADHD being the most common condition associated with TS, exhibiting a comorbidity rate of 60–80% in clinical settings [5]. However, the reasons behind the notably high comorbidity rate between TS and ADHD are currently not well-understood.

Symptoms of TS and ADHD exhibit overlaps on the clinical spectrum. On one hand, attentional deficits have been observed in TS patients [6–8], becoming more pronounced with comorbid ADHD [9]. On the other hand, they share a common feature: dysregulation of inhibition. Both conditions exhibit deficits in brain inhibitory control, often involving the inappropriate suppression of behaviors, thoughts, or emotional responses [10]. In the comorbidity of ADHD and TS, various neural network alterations are present, most notably within the cortico-striato-thalamo-cortical circuitry involved in tic generation and the impulsive characteristics of ADHD patients [5]. This circuitry also plays roles in planning, control, and executive functioning, affecting the sustainability of attentional focus in patients [5, 10]. A meta-analysis revealed that individuals with TS have mild to moderate inhibitory control deficits that worsen significantly with comorbid ADHD, indicating that these impairments are inherent to TS and amplified by ADHD [11]. However, previous studies have only identified overlaps in specific clinical features of ADHD and TS, without comprehensively assessing the characteristics of the associations between these features.

Historically, the development and examination of hypotheses concerning the complex interrelations of clinical symptoms have been challenging, largely due to the scarcity of suitable theories and computational methodologies [12]. Recent advancements in network analysis offer an array of promising solutions by depicting the intricate web of symptoms as graphical models where nodes (individual symptoms) are interconnected by edges (correlations and interactions) [13]. Such representations in network analysis offer a robust mathematical schema for exploring the intricate architecture of symptomatology. This analytical approach is particularly adept at pinpointing pivotal symptoms within the network, termed “core symptoms”, as well as those that serve as conduits between symptom clusters, known as “bridge symptoms” [13]. The concurrent manifestation of TS and ADHD symptoms has been associated with substantial detriments to an individual’s quality of life across various domains [14–17]. It is posited that core and bridge symptoms within this network could play critical roles in the emergence or persistence of the disorder. Consequently, precise identification and targeted intervention of these symptoms may greatly enhance therapeutic outcomes and improve the overall prognosis [18–20].

Thus, this study employed network analysis to investigate the interdependencies between individual TS and ADHD symptoms. Through this analysis, we aim to identify central indicators (the most core, influential symptoms within the comorbidity network) and bridge symptoms (transitional symptoms linking TS and ADHD), providing a theoretical basis for clinical treatment and intervention. We hypothesize that bridge symptoms exist between ADHD and TS, linking them together, and targeting these bridge symptoms for intervention could be one strategy to alleviate comorbidity.

## Methods

### Participants & procedure

This study recruited 4,148 children, from the pediatric psychiatry outpatient clinic, at Beijing Children’s Hospital from July 2023 to January 2024. Participants meeting the following criteria will be included in the study: (1) children or adolescents aged 6–16 years; (2) diagnosed with TS by child psychiatrists according to the Diagnostic and Statistical Manual of Mental Disorders, Fifth Edition (DSM-5), and assessed as having significant ADHD symptoms (diagnosed or subclinical ADHD) to capture a broader spectrum of ADHD symptoms. Additionally, participants will be excluded if they have other severe mental illnesses, such as intellectual disability, autism spectrum disorder and schizophrenia. Following the exclusion of samples with non-compliant questionnaire responses, the results yielded 3,958 valid samples for subsequent analysis. Guardians of all participants provided informed consent, and confidentiality regarding participants’ personal information was assured. The study received approval from the Ethics Committee of Beijing Children’s Hospital (Approval No. 2023-E-105-R).

### Measures

TS symptoms were assessed using the Motor Tic, Obsessions, and Compulsions, Vocal Tic Evaluation Survey (MOVES) [21]. MOVES is designed to measure the severity of tics and related sensory phenomena in TS, comprising 20 items rated on a 4-point scale from 0 (never) to 3 (always). It encompasses three primary dimensions: tic symptoms, obsessive-compulsive symptoms, and associated symptoms. Although it includes a dimension for obsessive-compulsive symptoms, in line with the original authors of MOVES, we consider it an assessment of thought processes related to TS in this study. MOVES is recognized for its good sensitivity and specificity for TS diagnosis and is recommended by the Committee on Rating Scale Development of the International Parkinson’s Disease and Movement Disorder Society as a screening tool for TS [22]. The MOVES demonstrates strong internal consistency, with Cronbach’s alpha values of 0.880 [23].

ADHD symptoms were evaluated using the Swanson, Nolan, and Pelham Rating Scale-IV (SNAP-IV), a tool adapted based on the Diagnostic and Statistical Manual of Mental Disorders, Fourth Edition (DSM-IV) criteria for screening and aiding in the diagnosis of ADHD [24]. The SNAP-IV consists of 18 items, rated from 0 (not at all) to 3 (very much), and includes two dimensions: “inattention” and “hyperactivity/impulsivity”. Subclinical ADHD is considered to be present when an individual meets at least six out of the nine items in the “inattention” domain and/or at least six out of the nine items in the “hyperactivity/impulsivity” domain of the SNAP-IV [24]. The SNAP-IV demonstrates strong internal consistency, with Cronbach’s alpha values ranging from 0.90 to 0.97 [25].

### Network analysis

All analyses were conducted in R version 4.3.3. Entries corresponding to TS or ADHD symptoms for each node indicator are detailed in Supplementary table S1.

#### Network construction

 This phase primarily utilized the qgraph package to build Gaussian graphical models (GGMs), analyzing the network relationships between TS and ADHD symptoms [26]. The process integrates L1 regularization (the graphical lasso, or glasso) with the extended Bayesian information criterion (EBIC) for model selection, producing a sparse network containing only strong Spearman correlations. This approach reduces the likelihood of spurious connections [27]. The Fruchterman-Reingold algorithm was employed for network layout, optimizing node positions to reflect the strength of relationships between them [28].

#### Relations between constructs

 The base and stats packages in R were used to calculate and compare relationships between symptoms. Following previous research [29], the number and proportion of non-zero edges between symptoms were first calculated to visually compare the quantity of connected edges between symptoms. Subsequently, non-parametric Wilcoxon tests or Kruskal-Wallis tests were performed to compare differences in edge weights between symptoms using the kruskal.test function and pairwise.wilcox.test function from the stats package.

#### Node centrality

 The qgraph and networktools packages in R were employed to calculate expected influence (EI) and bridge EI. EI is defined for a node as the sum of the absolute weights of the edges connecting it to other nodes in the network. EI denotes the importance of symptoms within the network [30], whereas bridge EI identifies which symptoms within one structure are most strongly associated with symptoms in another structure [31].

#### Network comparison

 The NetworkComparisonTest package in R was used to compare network invariance (distribution of edge weights) and global strength invariance (total absolute connectivity between symptoms) between two networks [32]. The NCT employs a two-tailed permutation testing approach, wherein differences between two distinct symptom networks are recalculated across multiple iterations with individuals randomly permuted [33]. Comparisons were conducted between male and female groups, as well as between child (under 10 years old) and adolescent (10 years old and above) groups, based on the definitions of developmental stages by the World Health Organization [34]. In addition, we verified whether there were differences in the network structure between the two cohorts: patients with TS and ADHD symptoms (including both diagnosed and subclinical cases), and those with both TS and ADHD diagnosis. M and S represent the differences in the mean values between network invariance and global strength invariance across two networks, respectively.

#### Network stability

 The bootnet package in R was utilized to assess the stability of the TS-ADHD network. Precision in edge weights was evaluated through 1,000 bootstrap resamplings, aimed at generating 95% confidence intervals for the distribution of edge weights. Network stability was quantified using the correlation stability (CS) coefficient obtained through the bootstrap person-drop procedure. A CS coefficient ≥ 0.25 indicates acceptable network stability, with a preferred value of ≥ 0.50, while a CS coefficient below 0.25 suggests inadequate network stability [35].

## Results

### Participant characteristics

A total of 3,958 participants were included in the final analysis, with an average age of 8.60 ± 2.25 years, comprising 3,004 males (76.9%). Further details are available in Table 1.

**Table 1:** Participant characteristics of participants (*n* = 3,958)

Variable	Value	
Sex, male, *n* (%)	3004	76.90
Age (years), mean (SD)	8.60	2.25
Diagnosis details, *n* (%)		
TS + ADHD	884	22.33
TS + Subclinical ADHD	3074	77.67
Drug-naive, *n* (%)	734	18.54
Psychotropic treatment, *n* (%)		
Clonidine	1224	30.92
Aripiprazole	1027	25.95
Atomoxetine	831	21.00
Sulpiride	187	4.72
Other	344	8.70
None	734	18.54
MOVES, mean (SD)		
MOVES total	11.29	8.81
MOVES tic symptoms total	5.98	4.35
MOVES obsessive-compulsive symptoms total	3.90	3.66
MOVES associated symptoms total	1.41	1.95
SNAP-IV, mean (SD)		
SNAP-IV total	18.44	10.87
SNAP-IV inattentive symptoms total	11.86	6.73
SNAP-IV hyperactive/impulsive symptoms total	6.58	5.32

MOVES: Motor tic, Obsessions and compulsions, Vocal tic Evaluation Survey; SNAP-IV: Swanson, Nolan, and Pelham Rating Scale-IV; SD: standard deviation

### Network construction

**Fig. 1:**
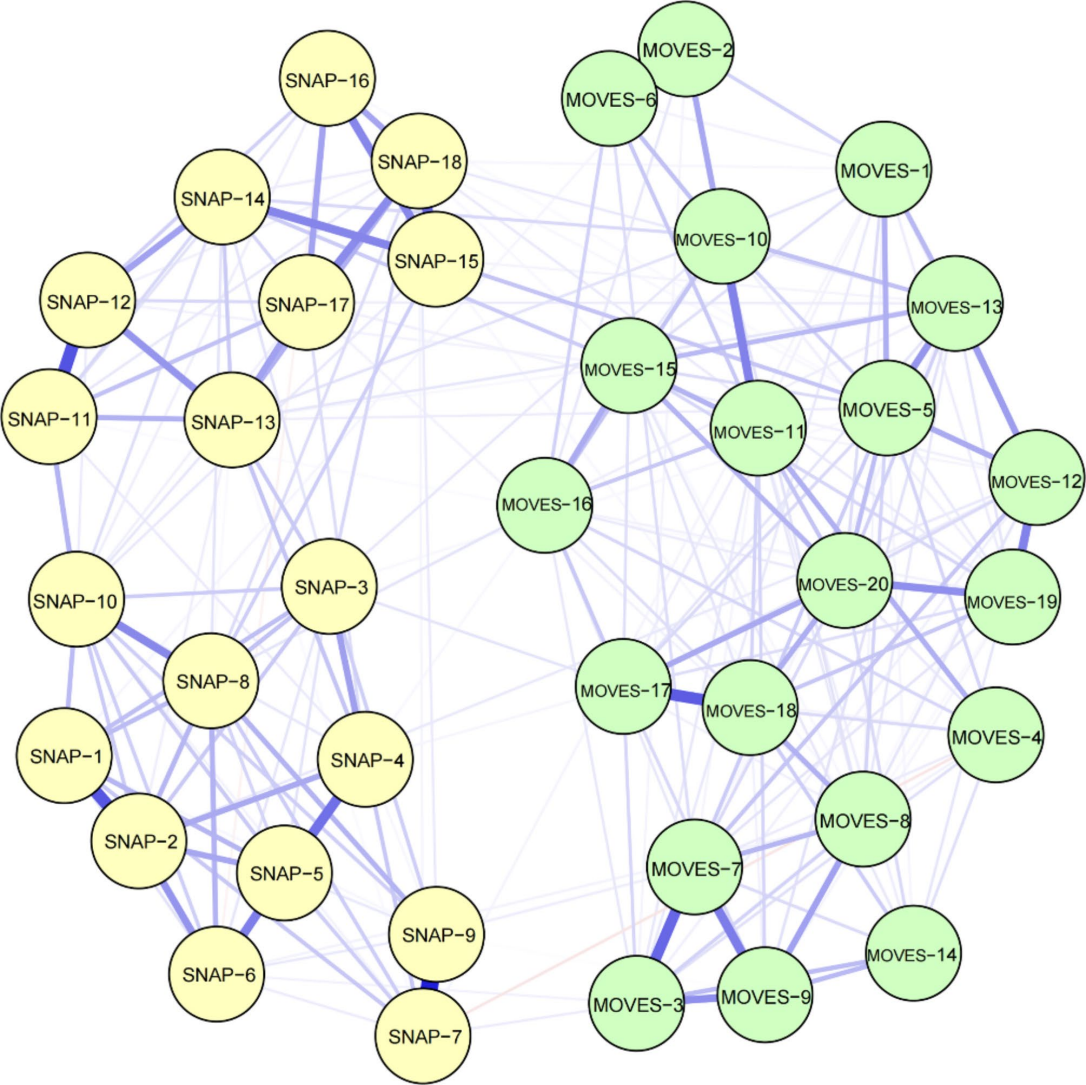
TS-ADHD comorbidity network structure. Green nodes represent TS symptoms, while yellow nodes indicate ADHD symptoms. The width of the connecting edges between symptoms signifies the strength of their association. Blue represents positive correlations, and red indicates negative correlations. Supplementary Figure S1 presents the same network, with nodes colored according to ADHD (inattentive symptoms, hyperactive/impulsive symptoms) and TS (tic symptoms, obsessive-compulsive symptoms, associated symptoms) dimensions. Supplementary Table S1 presents the specific meanings of SNAP-1 to SNAP-18 and MOVES-1 to MOVES-20. MOVES: Motor tic, Obsessions and compulsions, Vocal tic Evaluation Survey; SNAP-IV: Swanson, Nolan, and Pelham Rating Scale-IV

The network estimation identified 1,444 potential edges, of which 640 (44.32%) were non-zero, with an average weight of 0.05. Almost all edges were positive (*n* = 628, 98.13%), with a minimal number of negative edges (*n* = 12, 1.87%) (Fig. 1). No isolated nodes were observed within the network. Specifically, between any given TS symptom and ADHD symptom, there were 83 non-zero edges, accounting for 23.06% of all 360 possible edges. Between any TS symptom and inattentive symptoms, there were 42 non-zero edges (21.00% of all 200 possible edges), and between any TS symptom and hyperactive/impulsive symptoms, there were 41 non-zero edges (25.63% of all 160 possible edges). Between two ADHD symptoms, there were 36 non-zero edges (45% of 80 possible edges). Additionally, between two specific dimensions of TS symptoms (tic symptoms and obsessive-compulsive symptoms), there were 45 non-zero edges, making up 70.31% of all 64 possible edges.

A non-parametric Kruskal-Wallis test revealed significant differences in edge weights between TS-ADHD, ADHD-ADHD, and TS-TS symptoms (*χ²* = 44.373, *df* = 2, *p* < 0.001). Pairwise Wilcox tests for multiple comparisons showed that ADHD-ADHD connections were significantly stronger than TS-TS (*p* < 0.01) and ADHD-TS (*p* < 0.01) connections, and TS-TS connections were significantly stronger than ADHD-TS (*p* < 0.001). The Wilcoxon test comparing the differences in edge weights between the two groups revealed that connections between hyperactive/impulsive symptoms and TS (average edge weight = 0.017) were significantly stronger than those between inattentive symptoms and TS (average edge weight = 0.008) (W = 414, *p* < 0.05). There is no significant difference in the connections between vocal tics and ADHD symptoms (average edge weight = 0.010) and the connections between motor tics and ADHD symptoms (average edge weight = 0.009) (W = 115, *p* > 0.05).

### Node centrality

**Fig. 2:**
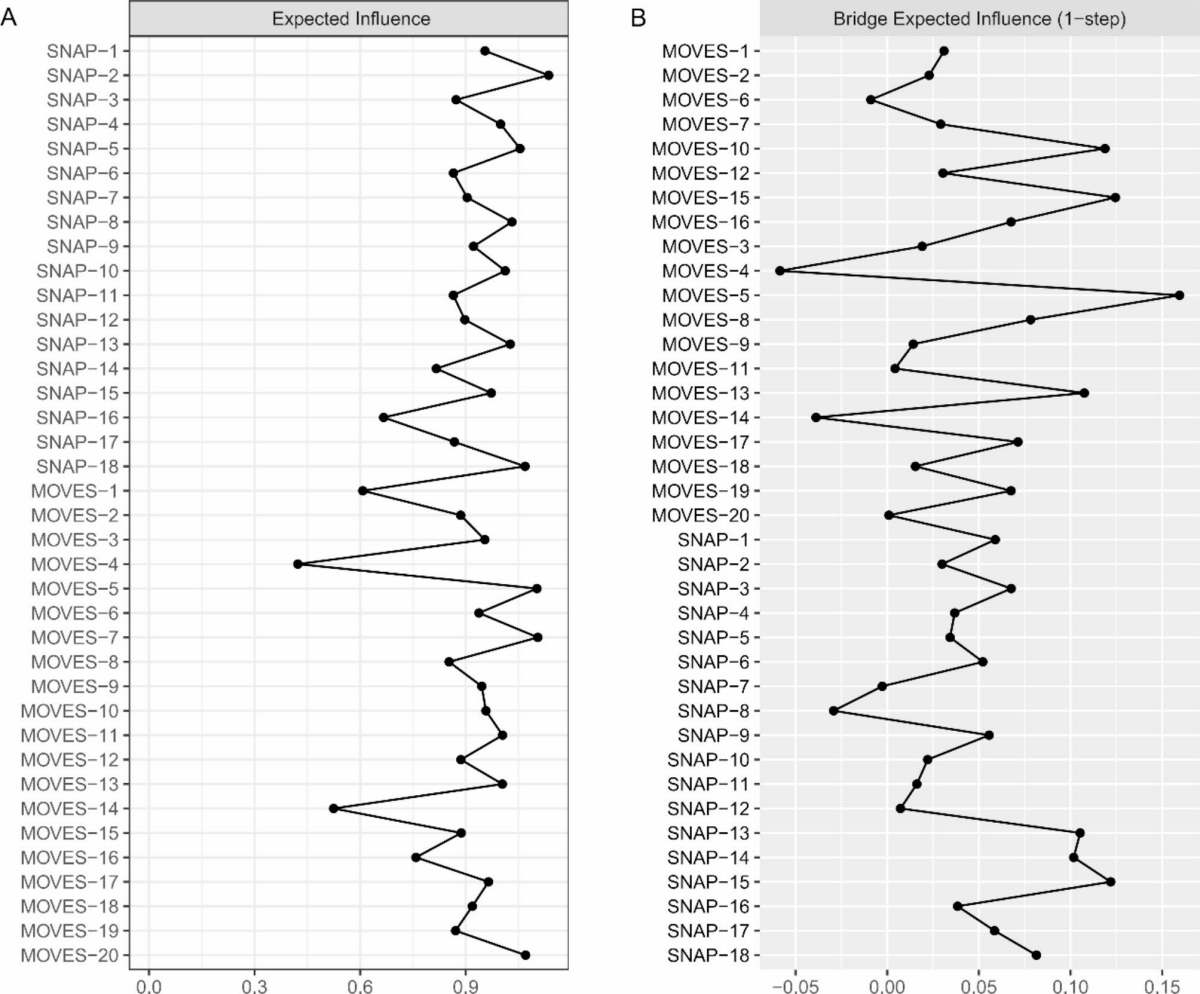
Expected influence (**A**) and bridge expected influence (**B**) within the TS-ADHD comorbidity network. Supplementary Table S1 presents the specific meanings of SNAP-1 to SNAP-18 and MOVES-1 to MOVES-20. MOVES: Motor tic, Obsessions and compulsions, Vocal tic Evaluation Survey; SNAP-IV: Swanson, Nolan, and Pelham Rating Scale-IV

To explore the key symptoms of the network, we utilized the expected influence (EI) and bridge EI metrics to explore the core and bridge symptoms within the network. The top five symptoms with the highest EI values are SNAP-2 “Often has difficulty sustaining attention in tasks or play activities” (EI = 1.48), MOVES-7 “Certain bad words or thoughts keep going through my mind” (EI = 1.28), MOVES-5 “Words come out that I can’t stop or control” (EI = 1.26), MOVES-20 “I have to repeat words or phrases over and over” (EI = 1.05), and SNAP-18 “Often interrupts or intrudes on others (e.g., butts into conversations/games)” (EI = 1.04) (Fig. 2A).

The five TS symptoms with the highest bridge EI values are MOVES-5 “Words come out that I can’t stop or control” (bridge EI = 0.16), MOVES-15 “I do certain things like jumping or clapping over and over” (bridge EI = 0.12), MOVES-10 “I can’t control all my movements” (bridge EI = 0.12), MOVES-13 “I feel pressure to talk, shout, or scream” (bridge EI = 0.11), and MOVES-8 “I have to do exactly the opposite of what I’m told” (bridge EI = 0.08). The five ADHD symptoms with the highest bridge EI values (indicating a strong association with TS) are SNAP-15 “Often talks excessively” (bridge EI = 0.12), SNAP-13 “Often has difficulty playing or engaging in leisure activities quietly” (bridge EI = 0.11), SNAP-14 “ ‘on the go’ or often acts as if ‘driven by a motor’ ” (bridge EI = 0.10), SNAP-18 “Often interrupts or intrudes on others (e.g., butts into conversations/games)” (bridge EI = 0.08), and SNAP-3 “Often does not seem to listen when spoken to directly” (bridge EI = 0.07) (Fig. 2B). Supplementary Tables S2 and S3 list the specific EI and bridge EI values for each item.

### Network comparison

First, to compare the network differences between the TS comorbid with ADHD group (*n* = 884) and the TS with a broad spectrum of ADHD symptoms group (diagnosed or subclinical ADHD, *n* = 3,958), we conducted a comparison of network structure and connectivity strength. The results showed that there was no significant difference in network structure between the two groups, as indicated by the network invariance test (M = 0.142, *p* = 0.680), meaning that the relationship patterns of TS symptoms and ADHD symptoms are consistent across both groups. However, according to the global strength invariance test (S = 1.085, *p* < 0.05), the overall connectivity strength of the TS with a broad spectrum of ADHD symptoms group (global strength: 18.017) was greater than that of the TS comorbid with ADHD group (global strength: 16.932). The specific network structure diagrams can be seen in supplementary Figure S2.

Then, to explore differences within the network between males and females, we conducted comparisons of network structure and connectivity strength. The total sample was divided based on gender into male (*n* = 3,004) and female (*n* = 954) subgroups for separate network constructions. The comparison revealed no significant differences in network structure between the two gender groups according to the network invariance test (M = 0.104, *p* = 0.680) and no significant differences in overall connectivity strength according to the global strength invariance test (S = 0.227, *p* = 0.610). The specific comorbidity network diagrams for males and females are presented in supplementary Figure S3.

Finally, to investigate differences in developmental stages within the network, we performed similar comparisons of network structure and connectivity strength. The total sample was divided by age into child (*n* = 2,730) and adolescent (*n* = 1,228) subgroups for independent network construction. Results showed no significant differences in network structure between the two developmental stage groups in the network invariance test (M = 0.111, *p* = 0.309), and no significant differences in overall connectivity strength in the global strength invariance test (S = 0.278, *p* = 0.372). The specific comorbidity network diagrams for children and adolescents are presented in supplementary Figure S4.

Thus, our results indicate no significant differences in network structure and overall connectivity strength between different sex groups, as well as between children and adolescent groups.

### Network Stability

Regarding network stability, both the EI and bridge EI centrality indices exhibited excellent stability, with an identical correlation stability (CS) coefficient of CS = 0.75 (95% CI: 0.675-1). This indicates that up to 75% of the sample could be omitted without causing significant changes in the network structure (supplementary Figure S5). The results of the bootstrap 95% confidence intervals (CI) for edge weights and bootstrap tests of differences in edge weights are displayed in supplementary Figure S6. The bootstrap difference tests revealed that most comparisons between edge weights were statistically significant (supplementary Figure S6).

## Discussion

This study is pioneering in applying network analysis to investigate the symptomatology relationship between TS and ADHD in a large clinical sample of children and adolescents. Overall, we identified a moderate number of non-zero connections (23.06% of all possible connections) between TS and ADHD symptoms within the network structure, indicating a definite symptomatic linkage between the two conditions. Notably, within the TS-ADHD symptom relationship network, often has difficulty staying focused (“Often has difficulty sustaining attention in tasks or play activities”), uncontrollably blurting out words (“Words come out that I can’t stop or control”) and an inability to control negative thoughts (“Certain bad words or thoughts keep going through my mind”) emerged as the most central symptoms. Additionally, uncontrollable words or actions (“Words come out that I can’t stop or control,” “I can’t control all my movements”), and excessive speaking or movements (“Often talks excessively,” “I do certain things like jumping or clapping over and over”), serve as bridge symptoms within the network.

Although our findings demonstrate some degree of connection between TS and ADHD symptoms, this relationship is not as pronounced as the high comorbidity rates observed clinically. We cautiously interpret this phenomenon, noting that during network construction, the graphical lasso (glasso) process, by regularizing weaker edge weights to zero, only retains more significant connections, thereby sacrificing sensitivity for specificity in network connections [27]. Connections within TS or ADHD symptoms were stronger than those between TS and ADHD, an expected outcome. Furthermore, the strength of overall connections between hyperactivity/impulsivity symptoms and TS was significantly stronger than that between inattention symptoms and TS, aligning with Heym and colleagues found that hyperactive/impulsive ADHD behaviors correlate positively with both pure TS behaviors and compulsive TS behaviors. In contrast, inattentive behaviors in ADHD show weaker or non-existent relationships with these TS behaviors [36].

The symptom “often has difficulty sustaining attention in tasks or play activities” is the most central within the TS-ADHD symptom relationship network, accompanied by significant symptoms such as uncontrollably blurting out words and an inability to control negative thoughts. In this comorbidity network, the most central symptom is persistent inattention. Despite some controversy, literature does indeed report occurrences of inattention within TS patients [6–8], which become more pronounced when ADHD is comorbid [9, 37]. This evidence suggests that inattention is the most significant and needing to be addressed symptom in comorbid patients. Besides, other symptoms involve control of maladaptive thinking (persistent thoughts) and behavior (incessant talking), underlying deficits in inhibitory control [10]. According to a meta-analysis, there is relatively reliable evidence for inhibitory control deficits in patients with comorbid TS and ADHD [11]. These inhibitory control deficits lead to a range of deficiencies in controlling behaviors, thoughts, or emotional responses [10].

Bridge symptoms include uncontrollable words or actions, and excessive speaking or movements. While research exploring the relationships between TS and ADHD symptoms at the symptomatic level is relatively scarce, our observation of the aforementioned crucial bridge symptoms reveals that they all pertain to dysregulation of behavioral control (difficulty controlling speech and actions), often underpinned by deficits in neuropsychological functions, predominantly inhibitory control [10]. In other words, it pertains to symptoms associated with dysregulation of behavioral control, linking these two manifestations together.

The relationship between TS and ADHD symptoms may not present significant variations across sex and developmental stages. Large-scale studies have observed differences in the prevalence of TS and ADHD between males and females [38, 39], and clinical diagnoses of TS in children are often accompanied by a higher incidence of ADHD comorbidity in late adolescence compared to healthy controls [40]. While various investigations have identified symptomatic differences across sex and developmental stages, research exploring how these factors influence the relationship between TS and ADHD symptoms is yet to be seen. Our network comparison results revealed no differences in the structure and overall strength of comorbidity networks between males and females, as well as between children and adolescents, suggesting that the symptomatic relationships and their intensities are consistent across the compared groups, indicating a relative stability in the relationship between TS and ADHD.

This study investigates core and bridge symptoms within the TS and ADHD symptom network, identifying them as key targets for intervention and treatment. Our findings offer important perspectives for clinical practice and contribute novel symptomatic evidence to understanding the complex interplay mechanisms between TS and ADHD symptoms, aiding in the development of more effective intervention measures and treatment strategies.

However, several limitations are present in this study. Firstly, although the study identified core and bridge symptoms within the comorbidity network, it only explored these on a symptomatic level, with the specific neural mechanisms behind these deficits requiring confirmation by future research. Secondly, being a cross-sectional study, it does not display longitudinal changes in the comorbidity network structure, necessitating further research for a comprehensive understanding. Third, this study was conducted solely within a clinical sample, limiting its broader applicability. Future research should aim to expand the sample, for instance, by further validation within community populations.

## Conclusion

In summary, this study employs network analysis techniques to identify core symptoms (inattention, uncontrollably blurting out words, and the inability to control negative thoughts) and bridge symptoms (uncontrollable words or actions, and excessive speaking or movements). These identified symptoms may serve as focal points for therapeutic strategies and interventions in clinical populations of children and adolescents with comorbid TS and ADHD symptoms. This provides a theoretical foundation for treatment and intervention approaches.

## Electronic supplementary material

Supplementary Material 1

## Data Availability

No datasets were generated or analysed during the current study.

## References

[1] Johnson KA, Worbe Y, Foote KD, Butson CR, Gunduz A, Okun MS. Tourette syndrome: clinical features, pathophysiology, and treatment. Lancet Neurol. 2023;22(2):147–58. 10.1016/s1474-4422(22)00303-9. 36354027 10.1016/S1474-4422(22)00303-9 PMC10958485

[2] Posner J, Polanczyk GV, Sonuga-Barke E. Attention-deficit hyperactivity disorder. Lancet. 2020;395(10222):450–62. 10.1016/s0140-6736(19)33004-1. 31982036 10.1016/S0140-6736(19)33004-1 PMC7880081

[3] Robertson MM, Eapen V, Singer HS, Martino D, Scharf JM, Paschou P, et al. Gilles De La Tourette syndrome. Nat Rev Dis Primers. 2017;3:16097. 10.1038/nrdp.2016.97. 28150698 10.1038/nrdp.2016.97

[4] Sayal K, Prasad V, Daley D, Ford T, Coghill D. ADHD in children and young people: prevalence, care pathways, and service provision. Lancet Psychiatry. 2018;5(2):175–86. 10.1016/s2215-0366(17)30167-0. 29033005 10.1016/S2215-0366(17)30167-0

[5] El Malhany N, Gulisano M, Rizzo R, Curatolo P. Tourette syndrome and comorbid ADHD: causes and consequences. Eur J Pediatr. 2015;174(3):279–88. 10.1007/s00431-014-2417-0. 25224657 10.1007/s00431-014-2417-0

[6] Chang SW, McCracken JT, Piacentini JC. Neurocognitive correlates of child obsessive compulsive disorder and Tourette syndrome. J Clin Exp Neuropsychol. 2007;29(7):724–33. 10.1080/13825580600966383. 17896198 10.1080/13825580600966383

[7] Harris EL, Schuerholz LJ, Singer HS, Reader MJ, Brown JE, Cox C, et al. Executive function in children with Tourette syndrome and/or attention deficit hyperactivity disorder. J Int Neuropsychol Soc. 1995;1(6):511–6. 10.1017/s1355617700000631. 9375237 10.1017/s1355617700000631

[8] Shucard DW, Benedict RH, Tekok-Kilic A, Lichter DG. Slowed reaction time during a continuous performance test in children with Tourette’s syndrome. Neuropsychology. 1997;11(1):147–55. 9055278 10.1037//0894-4105.11.1.147

[9] Sherman EM, Shepard L, Joschko M, Freeman RD. Sustained attention and impulsivity in children with Tourette syndrome: comorbidity and confounds. J Clin Exp Neuropsychol. 1998;20(5):644–57. 10.1076/jcen.20.5.644.1118. 10079041 10.1076/jcen.20.5.644.1118

[10] Sheppard DM, Bradshaw JL, Purcell R, Pantelis C. Tourette’s and comorbid syndromes: obsessive compulsive and attention deficit hyperactivity disorder. A common etiology? Clin Psychol Rev. 1999;19(5):531–52. 10.1016/s0272-7358(98)00059-2. 10467490 10.1016/s0272-7358(98)00059-2

[11] Morand-Beaulieu S, Grot S, Lavoie J, Leclerc JB, Luck D, Lavoie ME. The puzzling question of inhibitory control in Tourette syndrome: a meta-analysis. Neurosci Biobehav Rev. 2017;80:240–62. 10.1016/j.neubiorev.2017.05.006. 28502600 10.1016/j.neubiorev.2017.05.006

[12] McNally RJ. Network analysis of psychopathology: controversies and challenges. Annu Rev Clin Psychol. 2021;17:31–53. 10.1146/annurev-clinpsy-081219-092850. 33228401 10.1146/annurev-clinpsy-081219-092850

[13] Borsboom D, Deserno MK, Rhemtulla M, Epskamp S, Fried EI, McNally RJ, et al. Network analysis of multivariate data in psychological science. Nat Reviews Methods Primers. 2021;1(1):58. 10.1038/s43586-021-00055-w.

[14] Eapen V, Snedden C, Črnčec R, Pick A, Sachdev P. Tourette syndrome, co-morbidities and quality of life. Aust N Z J Psychiatry. 2016;50(1):82–93. 10.1177/0004867415594429. 26169656 10.1177/0004867415594429

[15] Ricketts EJ, Wolicki SB, Danielson ML, Rozenman M, McGuire JF, Piacentini J, et al. Academic, interpersonal, recreational, and family impairment in children with tourette syndrome and attention-deficit/hyperactivity disorder. Child Psychiatry Hum Dev. 2022;53(1):3–15. 10.1007/s10578-020-01111-4. 33385257 10.1007/s10578-020-01111-4 PMC8245573

[16] Eddy CM, Cavanna AE, Gulisano M, Calì P, Robertson MM, Rizzo R. The effects of comorbid obsessive-compulsive disorder and attention-deficit hyperactivity disorder on quality of life in tourette syndrome. J Neuropsychiatry Clin Neurosci. 2012;24(4):458–62. 10.1176/appi.neuropsych.11080181. 23224452 10.1176/appi.neuropsych.11080181

[17] Erbilgin Gün S, Kilincaslan A. Quality of life among children and adolescents with Tourette disorder and comorbid ADHD: a clinical controlled study. J Atten Disord. 2019;23(8):817–27. 10.1177/1087054718772158. 29707998 10.1177/1087054718772158

[18] Martel MM, Levinson CA, Langer JK, Nigg JT. A network analysis of developmental change in ADHD symptom structure from preschool to adulthood. Clin Psychol Sci. 2016;4(6):988–1001. 10.1177/2167702615618664. 28083448 10.1177/2167702615618664 PMC5222575

[19] Huang S, Lai X, Xue Y, Zhang C, Wang Y. A network analysis of problematic smartphone use symptoms in a student sample. J Behav Addict. 2020;9(4):1032–43. 10.1556/2006.2020.00098. 33372911 10.1556/2006.2020.00098 PMC8969737

[20] Cai H, Zhao YJ, He F, Li SY, Li ZL, Zhang WY, et al. Internet addiction and residual depressive symptoms among clinically stable adolescents with major psychiatric disorders during the COVID-19 pandemic: a network analysis perspective. Transl Psychiatry. 2023;13(1):186. 10.1038/s41398-023-02468-5. 37270593 10.1038/s41398-023-02468-5 PMC10238780

[21] Gaffney GS, Hellings K. The MOVES: a self-rating scale for Tourette’s syndrome. J Child Adolesc Psychopharmacol. 1994;4(4):269–80. 10.1089/cap.1994.4.269.

[22] Martino D, Pringsheim TM, Cavanna AE, Colosimo C, Hartmann A, Leckman JF, et al. Systematic review of severity scales and screening instruments for tics: critique and recommendations. Mov Disord. 2017;32(3):467–73. 10.1002/mds.26891. 28071825 10.1002/mds.26891 PMC5482361

[23] Zhang W, Wang X, Yang K, Zhang A, Yu L, Jiang Z, et al. Psychometric properties of the MOVES scale for Tourette syndrome and comorbidities in a Chinese cultural context. Child Psychiatry Hum Dev. 2024. 10.1007/s10578-024-01734-x. 38916698 10.1007/s10578-024-01734-x

[24] Gau SS, Shang CY, Liu SK, Lin CH, Swanson JM, Liu YC, et al. Psychometric properties of the Chinese version of the Swanson, Nolan, and Pelham, version IV scale - parent form. Int J Methods Psychiatr Res. 2008;17(1):35–44. 10.1002/mpr.237. 18286459 10.1002/mpr.237 PMC6878250

[25] Abhayaratna HC, Ariyasinghe DI, Ginige P, Chandradasa M, Hansika KS, Fernando A, et al. Psychometric properties of the Sinhala version of the Swanson, Nolan, and Pelham rating scale (SNAP-IV) parent form in healthy children and children with ADHD. Asian J Psychiatr. 2023;83:103542. 10.1016/j.ajp.2023.103542. 36963301 10.1016/j.ajp.2023.103542

[26] Epskamp S, Fried EI. A tutorial on regularized partial correlation networks. Psychol Methods. 2018;23(4):617–34. 10.1037/met0000167. 29595293 10.1037/met0000167

[27] Friedman J, Hastie T, Tibshirani R. Sparse inverse covariance estimation with the graphical lasso. Biostatistics. 2008;9(3):432–41. 10.1093/biostatistics/kxm045. 18079126 10.1093/biostatistics/kxm045 PMC3019769

[28] Fruchterman TMJ, Reingold EM. Graph drawing by force-directed placement. Software: Pract Exp. 1991;21:1129.

[29] Farhat LC, Brentani H, de Toledo VHC, Shephard E, Mattos P, Baron-Cohen S, et al. ADHD and autism symptoms in youth: a network analysis. J Child Psychol Psychiatry. 2022;63(2):143–51. 10.1111/jcpp.13436. 33984874 10.1111/jcpp.13436

[30] Robinaugh DJ, Millner AJ, McNally RJ. Identifying highly influential nodes in the complicated grief network. J Abnorm Psychol. 2016;125(6):747–57. 10.1037/abn0000181. 27505622 10.1037/abn0000181 PMC5060093

[31] Jones PJ, Ma R, McNally RJ. Bridge centrality: a network approach to understanding comorbidity. Multivar Behav Res. 2021;56(2):353–67. 10.1080/00273171.2019.1614898. 10.1080/00273171.2019.1614898 31179765

[32] van Borkulo CD, van Bork R, Boschloo L, Kossakowski JJ, Tio P, Schoevers RA, et al. Comparing network structures on three aspects: a permutation test. Psychol Methods. 2023;28(6):1273–85. 10.1037/met0000476. 35404628 10.1037/met0000476

[33] van Borkulo C, Boschloo L, Borsboom D, Penninx BW, Waldorp LJ, Schoevers RA. Association of Symptom Network structure with the course of [corrected] Depression. JAMA Psychiatry. 2015;72(12):1219–26. 10.1001/jamapsychiatry.2015.2079. 26561400 10.1001/jamapsychiatry.2015.2079

[34] Sawyer SM, Azzopardi PS, Wickremarathne D, Patton GC. The age of adolescence. Lancet Child Adolesc Health. 2018;2(3):223–8. 10.1016/s2352-4642(18)30022-1. 30169257 10.1016/S2352-4642(18)30022-1

[35] Epskamp S, Borsboom D, Fried EI. Estimating psychological networks and their accuracy: a tutorial paper. Behav Res Methods. 2018;50(1):195–212. 10.3758/s13428-017-0862-1. 28342071 10.3758/s13428-017-0862-1 PMC5809547

[36] Heym N, Kantini E, Checkley HL, Cassaday HJ. Tourette-like behaviors in the normal population are associated with hyperactive/impulsive ADHD-like behaviors but do not relate to deficits in conditioned inhibition or response inhibition. Front Psychol. 2014;5:946. 10.3389/fpsyg.2014.00946. 25228890 10.3389/fpsyg.2014.00946 PMC4151087

[37] Spencer T, Biederman J, Harding M, O’Donnell D, Wilens T, Faraone S, et al. Disentangling the overlap between Tourette’s disorder and ADHD. J Child Psychol Psychiatry. 1998;39(7):1037–44. 9804036

[38] Dy-Hollins ME, Chibnik LB, Tracy NA, Osiecki L, Budman CL, Cath DC, et al. Sex differences in people with Tourette syndrome and persistent motor or vocal tic disorder in the Tourette Association of America International consortium for genetics database. medRxiv. 2024. 10.1101/2024.01.07.24300816. 38260551 10.1101/2024.01.07.24300816 PMC10802652

[39] Garris J, Quigg M. The female Tourette patient: sex differences in Tourette disorder. Neurosci Biobehav Rev. 2021;129:261–8. 10.1016/j.neubiorev.2021.08.001. 34364945 10.1016/j.neubiorev.2021.08.001

[40] Gorman DA, Thompson N, Plessen KJ, Robertson MM, Leckman JF, Peterson BS. Psychosocial outcome and psychiatric comorbidity in older adolescents with Tourette syndrome: controlled study. Br J Psychiatry. 2010;197(1):36–44. 10.1192/bjp.bp.109.071050. 20592431 10.1192/bjp.bp.109.071050 PMC2894981

