# Supplementary material for: Network analysis of Tourette syndrome and attention-deficit/hyperactivity disorder symptoms in children and adolescents
Source: Child Adolesc Psychiatry Ment Health. 2024 Sep 16;18:118. doi: 10.1186/s13034-024-00810-3 (PMC11406714; doi:10.1186/s13034-024-00810-3)
Supplement: Supplementary file 1 — Supplementary Material 1 [file 13034_2024_810_MOESM1_ESM.docx]

**Supplementary Material**

**Table S1.** Content corresponding to labels in the TS-ADHD comorbidity network.

**Table S2.** Expected influence values of each item in the TS-ADHD comorbidity network.

**Table S3.** Bridge expected influence values of each item in the TS-ADHD comorbidity network.

**Figure S1.** TS-ADHD comorbidity network structure colored according to scale dimensions.

**Figure S2.** TS-ADHD comorbidity network structure of the patients diagnosed with both TS and ADHD according to the DSM-5.

**Figure S3.** TS-ADHD comorbidity network structure in male (A) and female (B).

**Figure S4.** TS-ADHD comorbidity network structure in children (A) and adolescents (B).

**Figure S5.** Centrality stability coefficients for expected influence (A) and bridge expected influence (B) within the TS-ADHD comorbidity network.

**Figure S6.** Nonparametric bootstrapped difference test.

**Table S1.** Content corresponding to labels in the TS-ADHD comorbidity network.

| Label | Item |
| --- | --- |
| MOVES-1 | I make noises (like grunts) that I can't stop. |
| MOVES-2 | Parts of my body jerk again and again, that I can't control. |
| MOVES-3 | I have bad ideas over and over, that I can't stop. |
| MOVES-4 | I have to do things in certain order or certain ways (like touching things). |
| MOVES-5 | Words come out that I can't stop or control. |
| MOVES-6 | At times I have the same jerk or twitch over and over. |
| MOVES-7 | Certain bad words or thoughts keep going through my mind. |
| MOVES-8 | I have to do exactly the opposite of what I'm told. |
| MOVES-9 | The same unpleasant or silly thought or picture goes through my mind. |
| MOVES-10 | I can't control all my movements. |
| MOVES-11 | I have to do several movements over and over again, in the same order. |
| MOVES-12 | Bad or swear words come out that I don't mean to say. |
| MOVES-13 | I feel pressure to talk, shout, or scream. |
| MOVES-14 | I have ideas that bother me (like germs or like cutting myself). |
| MOVES-15 | I do certain things (like jumping or clapping) over and over. |
| MOVES-16 | I have habits or movements that come out more when I'm nervous. |
| MOVES-17 | 1 have to repeat things that I hear other people say. |
| MOVES-18 | I have to do things I see other people do. |
| MOVES-19 | 1 have to make bad gestures (like the finger). |
| MOVES-20 | I have to repeat words or phrases over and over. |
| SNAP-1 | Often fails to give close attention to details or makes careless mistakes in schoolwork or tasks. |
| SNAP-2 | Often has difficulty sustaining attention in tasks or play activities. |
| SNAP-3 | Often does not seem to listen when spoken to directly. |
| SNAP-4 | Often does not follow through on instructions and fails to finish schoolwork, chores, or duties. |
| SNAP-5 | Often has difficulty organizing tasks and activities. |
| SNAP-6 | Often avoids, dislikes, or reluctantly engages in tasks requiring sustained mental effort. |
| SNAP-7 | Often loses things necessary for activities (e.g., toys, school assignments, pencils or books). |
| SNAP-8 | Often is distracted by extraneous stimuli. |
| SNAP-9 | Often is forgetful in daily activities. |
| SNAP-10 | Often fidgets with hands or feet or squirms in seat. |
| SNAP-11 | Often leaves seat in classroom or in other situations in which remaining seated is expected. |
| SNAP-12 | Often runs about or climbs excessively in situations in which it is inappropriate. |
| SNAP-13 | Often has difficulty playing or engaging in leisure activities quietly. |
| SNAP-14 | Often is “on the go” or often acts as if “driven by a motor”. |
| SNAP-15 | Often talks excessively. |
| SNAP-16 | Often blurts out answers before questions have been completed. |
| SNAP-17 | Often has difficulty awaiting turn. |
| SNAP-18 | Often interrupts or intrudes on others (e.g., butts into conversations/games). |

TS, Tourette syndrome; ADHD, attention-deficit/hyperactivity disorder; MOVES: Motor tic, Obsessions and compulsions, Vocal tic Evaluation Survey; SNAP-IV: Swanson, Nolan, and Pelham Rating Scale-IV.

**Table S2.** Expected influence values of each item in the TS-ADHD comorbidity network.

| Node | Value |
| --- | --- |
| MOVES-1 | -1.986846565 |
| MOVES-2 | -0.161955601 |
| MOVES-6 | 0.179507539 |
| MOVES-7 | 1.27716133 |
| MOVES-10 | 0.308150272 |
| MOVES-12 | -0.157893241 |
| MOVES-15 | -0.151799743 |
| MOVES-16 | -0.996129921 |
| MOVES-3 | 0.288037801 |
| MOVES-4 | -3.197378964 |
| MOVES-5 | 1.259931067 |
| MOVES-8 | -0.378638319 |
| MOVES-9 | 0.231155918 |
| MOVES-11 | 0.618761009 |
| MOVES-13 | 0.614883946 |
| MOVES-14 | -2.531085525 |
| MOVES-17 | 0.357473319 |
| MOVES-18 | 0.055490542 |
| MOVES-19 | -0.257420436 |
| MOVES-20 | 1.048001832 |
| SNAP-1 | 0.290768722 |
| SNAP-2 | 1.477774485 |
| SNAP-3 | -0.247678791 |
| SNAP-4 | 0.582523491 |
| SNAP-5 | 0.945442876 |
| SNAP-6 | -0.296678424 |
| SNAP-7 | -0.042467338 |
| SNAP-8 | 0.793530758 |
| SNAP-9 | 0.077861095 |
| SNAP-10 | 0.669234418 |
| SNAP-11 | -0.298793408 |
| SNAP-12 | -0.084821641 |
| SNAP-13 | 0.762976116 |
| SNAP-14 | -0.615708362 |
| SNAP-15 | 0.407816261 |
| SNAP-16 | -1.59953266 |
| SNAP-17 | -0.279620522 |
| SNAP-18 | 1.037966667 |

TS, Tourette syndrome; ADHD, attention-deficit/hyperactivity disorder; MOVES: Motor tic, Obsessions and compulsions, Vocal tic Evaluation Survey; SNAP-IV: Swanson, Nolan, and Pelham Rating Scale-IV.

**Table S3.** Bridge expected influence values of each item in the TS-ADHD comorbidity network.

| Node | Value |
| --- | --- |
| MOVES-1 | 0.031002655 |
| MOVES-2 | 0.022840805 |
| MOVES-6 | -0.008909816 |
| MOVES-7 | 0.029188469 |
| MOVES-10 | 0.118824765 |
| MOVES-12 | 0.030396669 |
| MOVES-15 | 0.124451973 |
| MOVES-16 | 0.067573102 |
| MOVES-3 | 0.019072275 |
| MOVES-4 | -0.058496823 |
| MOVES-5 | 0.159440834 |
| MOVES-8 | 0.078224815 |
| MOVES-9 | 0.014203527 |
| MOVES-11 | 0.00426918 |
| MOVES-13 | 0.107512093 |
| MOVES-14 | -0.038836353 |
| MOVES-17 | 0.071329157 |
| MOVES-18 | 0.015396722 |
| MOVES-19 | 0.067539313 |
| MOVES-20 | 0.000940634 |
| SNAP-1 | 0.058946589 |
| SNAP-2 | 0.029913672 |
| SNAP-3 | 0.067623673 |
| SNAP-4 | 0.036777065 |
| SNAP-5 | 0.03427707 |
| SNAP-6 | 0.052181736 |
| SNAP-7 | -0.002707459 |
| SNAP-8 | -0.029214485 |
| SNAP-9 | 0.055598648 |
| SNAP-10 | 0.022081859 |
| SNAP-11 | 0.016168462 |
| SNAP-12 | 0.00724497 |
| SNAP-13 | 0.105228669 |
| SNAP-14 | 0.101710031 |
| SNAP-15 | 0.121869505 |
| SNAP-16 | 0.038392456 |
| SNAP-17 | 0.058505804 |
| SNAP-18 | 0.081365726 |

TS, Tourette syndrome; ADHD, attention-deficit/hyperactivity disorder; MOVES: Motor tic, Obsessions and compulsions, Vocal tic Evaluation Survey; SNAP-IV: Swanson, Nolan, and Pelham Rating Scale-IV.


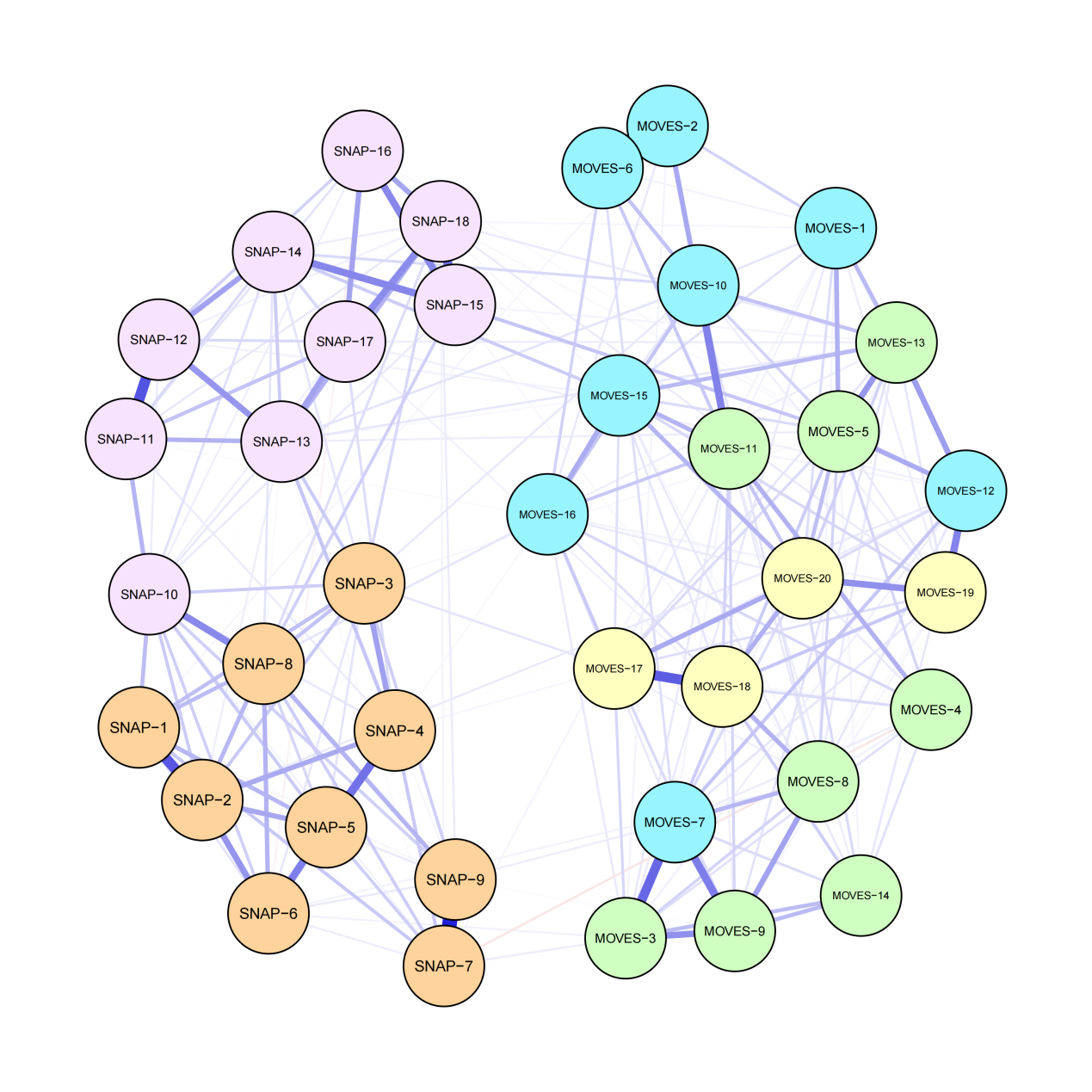


**Figure S1.** TS-ADHD comorbidity network structure colored according to scale dimensions. TS, Tourette syndrome; ADHD, attention-deficit/hyperactivity disorder; MOVES: Motor tic, Obsessions and compulsions, Vocal tic Evaluation Survey; SNAP-IV: Swanson, Nolan, and Pelham Rating Scale-IV.


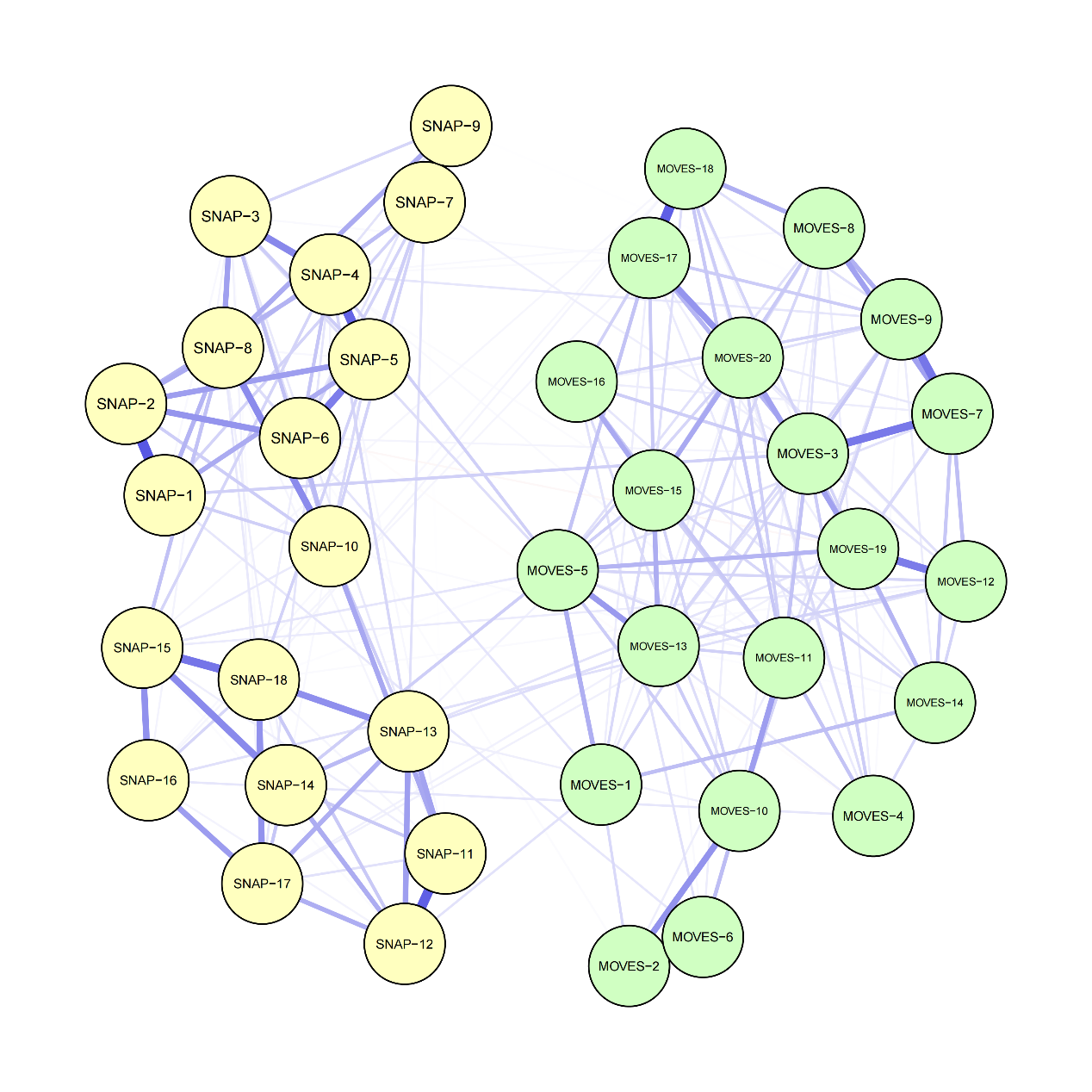


**Figure S2.** TS-ADHD comorbidity network structure of the patients diagnosed with both TS and ADHD according to the DSM-5. TS, Tourette syndrome; ADHD, attention-deficit/hyperactivity disorder; MOVES: Motor tic, Obsessions and compulsions, Vocal tic Evaluation Survey; SNAP-IV: Swanson, Nolan, and Pelham Rating Scale-IV.


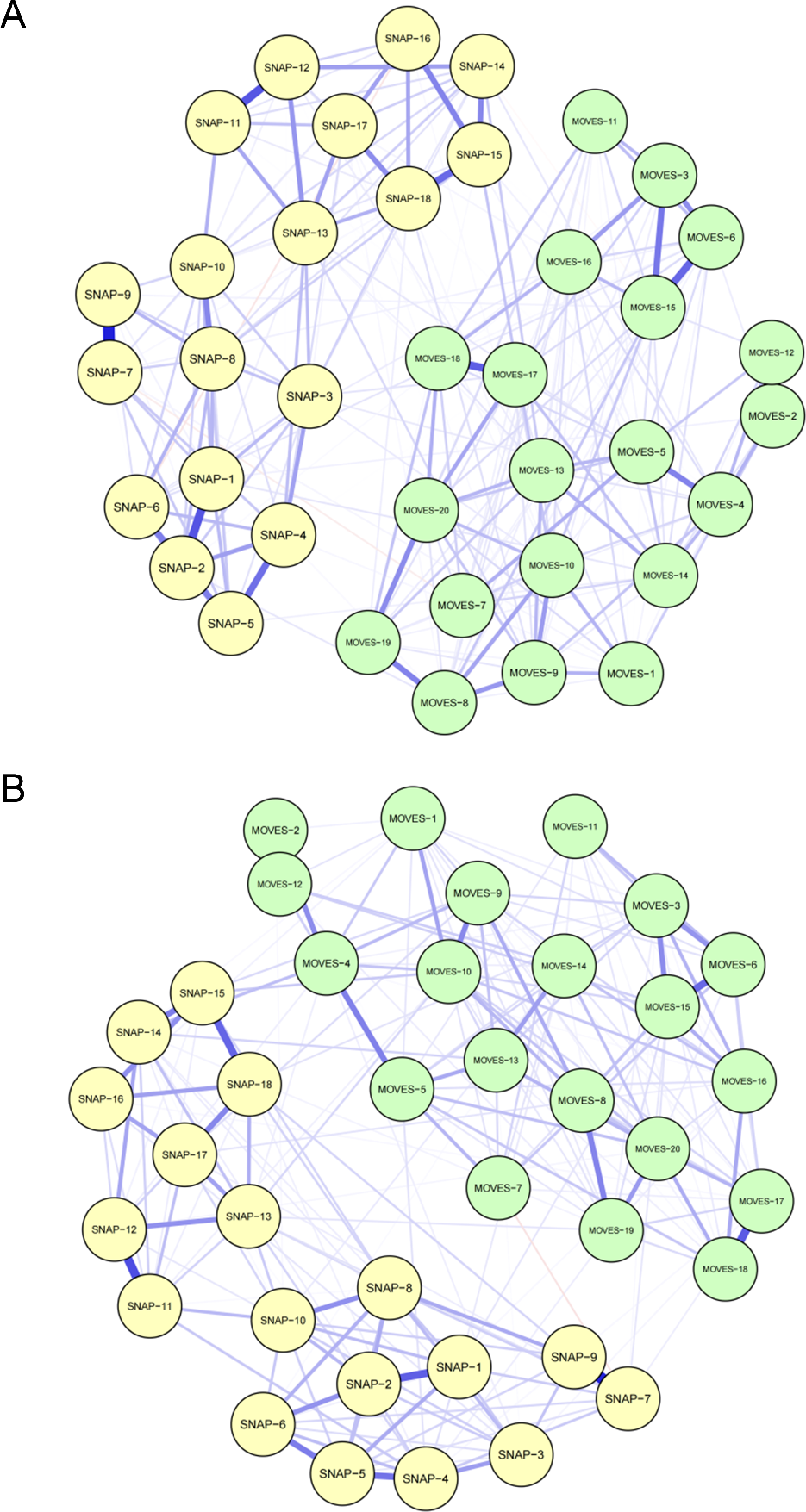


**Figure S3.** TS-ADHD comorbidity network structure in male (A) and female (B). TS, Tourette syndrome; ADHD, attention-deficit/hyperactivity disorder; MOVES: Motor tic, Obsessions and compulsions, Vocal tic Evaluation Survey; SNAP-IV: Swanson, Nolan, and Pelham Rating Scale-IV.


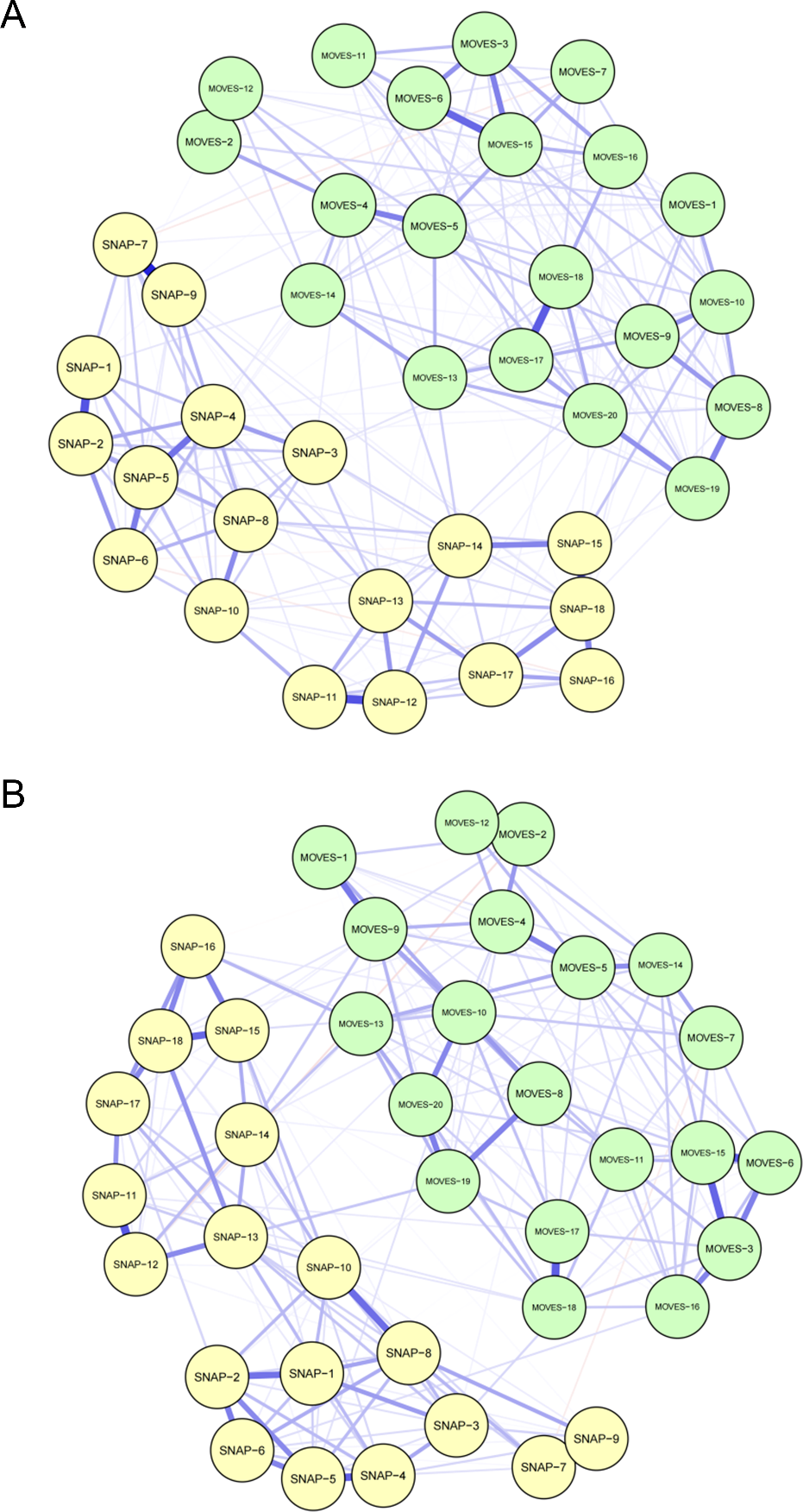


**Figure S4.** TS-ADHD comorbidity network structure in children (A) and adolescents (B). TS, Tourette syndrome; ADHD, attention-deficit/hyperactivity disorder; MOVES, the Motor Tic, Obsessions and Compulsions, Vocal Tic Evaluation Survey; SNAP, the Swanson, Nolan, and Pelham Rating Scale-IV.


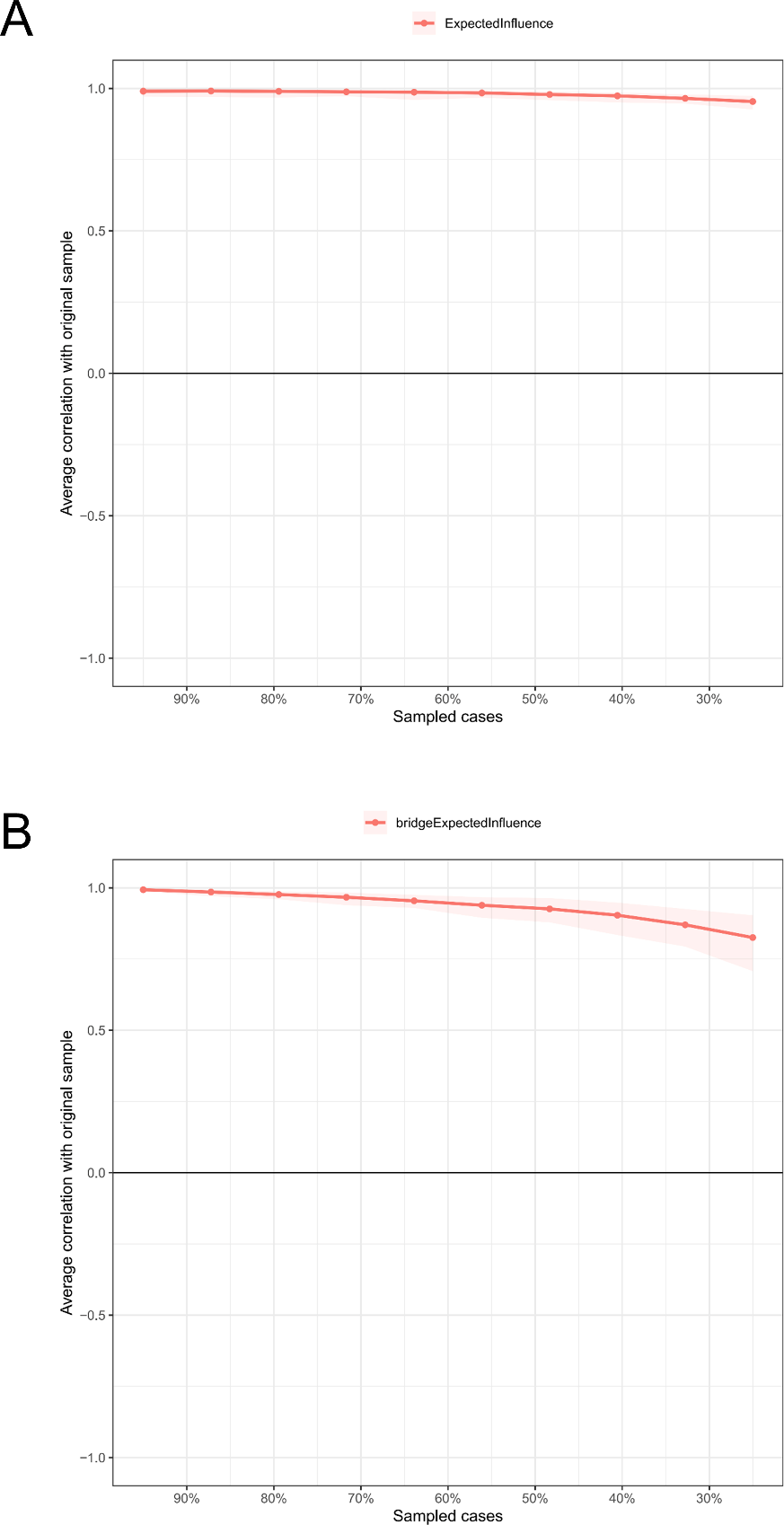


**Figure S5.** Centrality stability coefficients for expected influence (A) and bridge expected

influence (B) within the TS-ADHD comorbidity network.


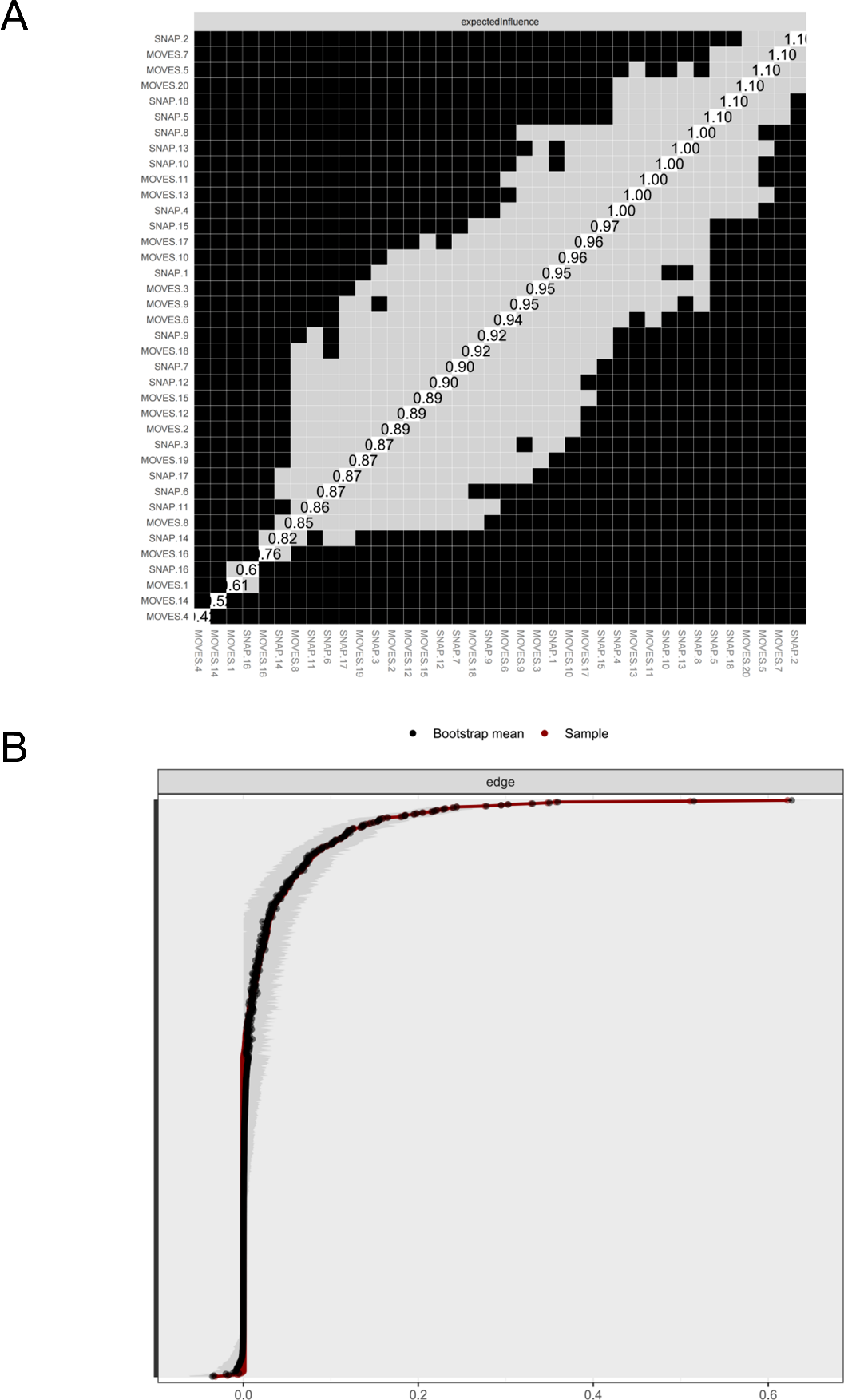


**Figure S6.** Nonparametric bootstrapped difference test. (A) Difference test for node degree centrality. (B) Accuracy of edge weights.
